# The Omission of Anthracycline Chemotherapy in Women with Early HER2-Negative Breast Cancer—A Systematic Review and Meta-Analysis

**DOI:** 10.3390/curroncol31080335

**Published:** 2024-08-03

**Authors:** Danilo Giffoni de Mello Morais Mata, Mary-Beth Rush, Megan Smith-Uffen, Jawaid Younus, Ana Elisa Lohmann, Maureen Trudeau, Rebecca L. Morgan

**Affiliations:** 1 Division of Medical Oncology, Verspeeten Family Cancer Centre, London Health Sciences Centre, London, ON N6A 5W9, Canada; jawaid.younus@lhsc.on.ca (J.Y.); ana.lohmann@lhsc.on.ca (A.E.L.); 2 Department of Medicine, Schulich School of Medicine and Dentistry, Western University, London, ON N6A 5C1, Canada; 3 Department of Health Research Methods, Evidence and Impact (HEI), Faculty of Health Sciences, McMaster University, Hamilton, ON L8N 3Z5, Canada; mary-beth.rush@sinaihealth.ca; 4 Department of Medicine, Faculty of Health Sciences, McMaster University, Hamilton, ON L8N 3Z5, Canada; megan.smithuffen@medportal.ca; 5 Division of Medical Oncology, Odette Cancer Centre, Sunnybrook Health Sciences Centre, Toronto, ON M4N 3M5, Canada; maureen.trudeau@sunnybrook.ca; 6 Department of Medicine, University of Toronto, Toronto, ON M5S 1A1, Canada; 7 School of Medicine, Case Western Reserve University, Cleveland, OH 44106, USA

**Keywords:** human epidermal growth factor receptor 2 negative, endocrine (hormone) receptor, paclitaxel, docetaxel, cyclophosphamide, doxorubicin, epirubicin, adjuvant, invasive carcinoma

## Abstract

Background: Anthracycline-taxane is the standard chemotherapy strategy for treating high-risk early breast cancer despite the potentially life-threatening adverse events caused by anthracyclines. Commonly, the combination of docetaxel and cyclophosphamide (TC) is considered an alternative option. However, the efficacy of TC compared to anthracycline-taxane chemotherapy is unclear. This study compares disease-free survival (DFS), overall survival (OS) and cardiotoxicity between adjuvant TC and anthracycline-taxane for stages I–III, HER2-negative breast cancer. Methods: A systematic search on MEDLINE, Embase and Cochrane CENTRAL for randomized-controlled trials published until 11 March 2024, yielded 203 studies with 11,803 patients, and seven trials were included. Results: TC results in little to no difference in DFS (HR 1.09, 95% CI 0.98–1.20; moderate-certainty of evidence); OS (1.02, 95% CI 0.89–1.16; high-certainty of evidence); and cardiotoxicity (RR 0.54, 95% CI 0.16–1.76; high-certainty of evidence), compared to anthracycline-taxane. In the subgroup analysis, patients with ≥4 lymph nodes had improved DFS from anthracycline-taxane over TC. Conclusions: Overall, there was no difference between TC and anthracycline-taxane in DFS, OS and cardiotoxicity. In women with ≥4 nodes, anthracycline-taxane was associated with a substantial reduction in relapse events, compared to TC. Our study supports the current standard of practice, which is to use anthracycline-taxane and TC chemotherapy as a reasonable option in select cases.

## 1. Introduction

Breast cancer is the most common oncological condition among women, accounting for 25% of all new cancer cases [1]. At the moment of diagnosis, approximately 95% of patients have stage I–III breast cancer, and the vast majority are eligible for curative treatment approaches [2,3]. In these cases, early tumor detection and novel strategies to tailor optimal therapy have steadily enhanced breast cancer-specific survival, resulting in a 90.8% 5-year survival [4,5,6].

Three out of four breast cancer patients will have tumors expressing luminal differentiation with an endocrine receptor (ER)-positive subtype [7]. In these cases, postoperative treatment options include chemotherapy, endocrine therapy (ET) plus/minus monoclonal antibodies against the human epidermal growth factor receptor 2 (HER2) and radiation [8,9,10]. Adjuvant systemic treatments aim to eliminate micrometastatic cells undetected in the surgical specimen [10]. In stage I–III HER2-negative breast cancer, adjuvant chemotherapy is the treatment of choice when pathology or genomic expression assays indicate a high risk for cancer relapse [11].

Predictive factors of chemotherapy efficacy are often associated with ER and HER2 positivity, high-risk scores on genomic profiling assays and breast cancer morphological features [12,13,14]. For example, poorly differentiated histology, luminal B and basal-like triple-negative breast cancer (TNBC), as well as evidence of high tumor pathogenesis such as lymph node dissemination and inflammatory carcinoma, are susceptible to increased chemotherapy responsiveness [15,16,17]. Conversely, breast tumors, typically luminal A with overexpression of ER, lobular and mucinous morphological phenotypes, have low chemotherapy effectiveness, likely due to their increased endocrine treatment sensitivity [18,19].

Over the past three decades, the use of anthracyclines has become a cornerstone in the treatment of breast cancer [20]. The subsequent addition of taxanes to anthracyclines has further improved recurrence risk, overall survival (OS) and breast cancer-specific mortality [21,22]. Unfortunately, anthracyclines have a myriad of adverse effects. In addition to carrying high emetogenic and myelosuppressive potential, they can cause irreversible congestive heart failure (CHF) [23,24].

Anthracycline-induced CHF occurs between 6% and 13% after exposure, with an HR of 3.25 (95% CI, 2.11–5.00); *p* < 0.001) [25]. Underlying cardiac comorbidity and the usage of anti-HER2 therapies, such as trastuzumab and pertuzumab, elevate the risk of cardiac-related events [25,26]. Notably, in patients treated with versus without anthracyclines, the cumulative incidence of CHF likelihood rises over time, and its increased absolute risk from treatment completion is 1.72% at 1 year, 2.12% at 5 years, 3.62% at 10 years and 5.67% at the 25-year mark (*p*  <  0.001) [26]. Though a late onset is typically subclinical, but can persist long after treatment is completed, causing debilitating disease [23,24].

Although anthracycline-taxane chemotherapy remains the keystone option for breast cancer harboring high-risk features, in many cases, non-anthracycline regimens are considered a reasonable alternative in select populations. For example, in patients with cardiac conditions, previous exposure to anthracyclines and those who are at high risk for breast cancer recurrence, a doublet platinum taxane chemotherapy has shown similar outcomes to those of anthracycline-taxane in the treatment of TNBC- and HER2-positive diseases [27,28,29]. Furthermore, in women who have intermediate-risk breast cancer, the docetaxel and cyclophosphamide (TC) regimen, which combines a taxane with an alkylating agent, is often selected due to better tolerability [30]. TC also has the advantage of shorter treatment duration, requiring fewer visits to the cancer center [31].

Although TC has been associated with fewer cardiotoxicity events in some studies [32], as compared with anthracyclines, in many trials, there was a small number of cardiac events overall, making it difficult to determine a significant difference between the two regimens [33]. Several randomized controlled trials (RCTs) demonstrated that TC is non-inferior to anthracycline-based chemotherapy [34,35]. However, most of these studies consisted of an omission of a taxane component, lacking comparison with the standard anthracycline-taxane regimens.

While many molecular drivers and gene mutations, such as PDL-1 positivity, tumor-infiltrating lymphocyte (TIL) and germline BRCA carriage, do not appear to derive substantial benefit from anthracyclines, it remains unclear which specific tumor features and breast cancer populations would benefit the most from TC over anthracycline-taxane chemotherapy [36,37,38].

To our knowledge, there has not yet been an updated systematic review or meta-analysis on relevant RCTs published after February 2019 [39] that compares recurrence and survival-related endpoints between TC and anthracycline-taxane-based chemotherapy across all randomized trials in women with HER2-negative breast cancer. Our study is the first systematic review and meta-analysis to compare cardiotoxicity between anthracycline-taxane versus non-anthracycline regimens in breast cancer without the use of monoclonal antibodies against the HER2 domain.

We reviewed the existing literature comparing TC and anthracycline-taxane-containing chemotherapy regimens in patients with stages I–III HER2-negative breast cancer to investigate and compare their impact on DFS, OS and cardiotoxicity.

## 2. Materials and Methods

This research protocol was registered with PROSPERO (registration number: CRD42022326695) [40], and this study was conducted in accordance with the Preferred Reporting Items for Systematic Reviews and Meta-Analyses (PRISMA) 2020 statement [41].

### 2.1. Search Strategy

We searched Ovid Medline (1946–2024), EMBASE (1974–2024) and Evidence-Based Medicine Reviews: Cochrane Central Register of Controlled Trials (1991–2024) and ClinicalTrials.gov for RCTs comparing TC to anthracycline-taxane-based chemotherapy for adult patients with histologically confirmed HER2-negative, stages I–III breast cancer. Studies needed to include DFS and OS and be published in English. The literature search included trials published until 11 March 2024.

We also reviewed the reference lists of the relevant literature reviews and articles. We searched trial websites and databases, such as ClinicalTrials.gov, the World Health Organization (WHO) International Clinical Trials Registry Platform, as well as International Standard Randomised Controlled Trial Number (ISRCTN) for additional publication information. The following material was excluded from our review: book chapters, case reports, editorial letters, review articles, retrospective non-randomized studies, single-arm trials and opinion papers.

### 2.2. Selection of Studies

Two authors (DG and MR) independently screened the titles and abstracts of all search results using the web-based platform Covidence^®^ [42]. Disagreements about study inclusion were resolved through discussion.

### 2.3. Data Extraction and Quality Assessment

Two authors (DG and MR) independently extracted data using a standardized form. The following information was collected for each included study: participant’s eligibility criteria, trial design and objectives, characteristics of the intervention and comparison groups, including information about treatment and follow-up duration, analysis methods, study outcomes and missing data. Studies that included multiple trials had data extracted based on each trial and their respective trial protocols.

### 2.4. Outcome Measures

DFS is defined as the time from randomization to the development of breast cancer recurrence or death [43]. OS was defined as the time from randomization until death from any cause [43]. Cardiotoxicity was defined as participants who developed signs and symptoms of heart failure (HF) or left ventricular systolic dysfunction (LVSD) in the context of recent or remote chemotherapy administration [44]. Only grade 3 or above cardiotoxic adverse events were extracted, as measured by the Common Terminology Criteria for Adverse Events (CTCAE) v5.0 standardized grading system [45].

### 2.5. Statistical Analysis

All analyses were performed using Review Manager software, version 5.4. We pooled data based on the generic inverse variance method for hazard ratios (HRs) to evaluate DFS and OS. Treatment effects were reported as HR with 95% confidence intervals (CIs). HR less than 1.0 was reported to favor TC chemotherapy, and those greater than 1.0 to favor anthracycline-taxane-containing chemotherapy. We expected heterogeneity among clinical, methodological and population characteristics and therefore chose a random-effects model. A subgroup analysis of DFS and OS based on ER and lymph node status was conducted.

Cardiotoxicity as a dichotomous outcome was reported as a risk ratio (RR) using the Mantel–Haenszel method. An RR of less than 1 favored TC regimens and greater than 1 favored anthracycline-based regimens. The number of cardiotoxicity events was based on the number of patients who started treatment within each study. The number of events was pooled with participants who experienced HF or LVSD during the follow-up period. We emailed one author to evaluate missing data regarding cardiotoxicity [46]. However, no response was received. In trials with multiple arms, only the arms of relevance to the review were included in the analysis.

### 2.6. Quality Assessment

#### 2.6.1. Risk of Bias

The risk of bias (RoB) was assessed by two reviewers (DG and MR), who independently assessed all articles using the Cochrane RoB 2.0 tool for RCTs. The domains assessed included the randomization process, deviations from intended interventions, missing outcome data, outcome measurement and selection of the reported results [47]. We used the Revised Cochrane risk-of-bias tool for randomized trials (RoB2) to guide risk evaluation for each outcome of interest [48]. We chose this tool as the studies were individually randomized parallel trials. Domains were given ratings of “low risk”, “some concerns” or “high risk” by each reviewer, followed by an overall risk judgment for the outcome [47]. Discrepancies were resolved through discussion.

#### 2.6.2. Assessment of Heterogeneity

Heterogeneity was assessed using I^2^ and Chi^2^ statistics, as well as visual inspection of the forest plots for alignment of the point estimates and the extent of overlapping CIs. We interpreted the I^2^ statistic based on the following: the heterogeneity “might not be important” (0% to 40%), “may be moderate” (30% to 60%), “may be substantial” (50% to 90%) or “considerable” (75% to 100%). A *p*-value of less than 0.10 for the Chi^2^ test was considered significant heterogeneity [47].

#### 2.6.3. Assessment of Reporting Biases

Funnel plots were not generated to assess for reporting bias as there were fewer than 10 studies. Reporting bias was assessed by examining study protocols when available.

#### 2.6.4. Sensitivity Analysis

We performed a RoB sensitivity analysis to examine the effects of high/moderate risk studies on each outcome. High RoB was defined as studies with poor randomization or allocation concealment descriptions and selective reporting. Studies were not excluded in the sensitivity analysis based on a lack of pre-specified data analysis plans, as it did not seem to affect their reporting of the results.

#### 2.6.5. Assessment of Certainty of Evidence

Two reviewers (DG and MR) assessed the certainty of the evidence for the outcomes of DFS, OS and cardiotoxicity as high, moderate, low or very low, as per the GRADE criteria [49]. We used the domains RoB, inconsistency, indirectness, imprecision and publication bias to assess the certainty of the evidence for each of the evidence for reported outcomes [49,50].

## 3. Results

### 3.1. Search Selection

**Figure 1:**
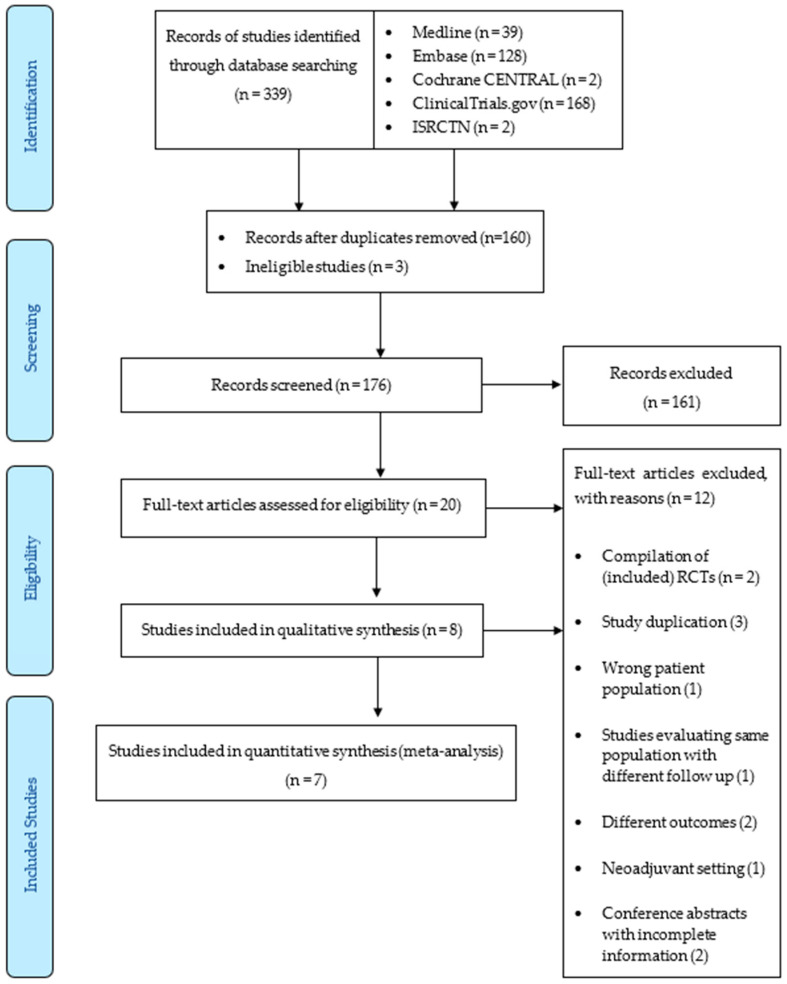
Study flow PRISMA diagram.

Our search yielded 203 published studies from which 8 RCTs were included in the qualitative analysis and 7 RCTs in the quantitative (meta) analysis [33,46,51,52,53,54,55] (Figure 1). In total, 11,803 women were included. Two studies reported a meta-analysis of 3 trials [46,55], which we analyzed separately. Another study was a pooled meta-analysis of two separate trials (SUCCESS-C and Plan B) [46,54]. Only the SUCCESS-C data were utilized, as the Plan B data were previously published. Characteristics of the included trials can be found in Table 1.

### 3.2. Assessment of the Evidence

#### 3.2.1. Risk of Bias

**Figure 2:**
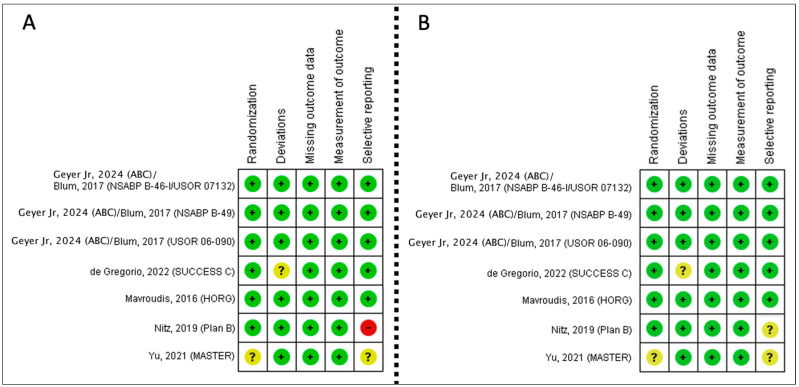
(**A**) Risk of bias summary by domain for disease-free survival. (**B**) Risk of bias summary by domain for overall survival [33,46,51,52,54,55] (color should be used in print version of these figures).

There are seven trials included in the RoB assessment for DFS (Figure 2A) and seven for OS (Figure 2B).

#### 3.2.2. Randomization

All studies, except for one, described sufficient processes for both randomization and allocation concealment [33]. This study stated that patients were randomized; however, it did not explain the process for either allocation concealment or randomization. Most studies included their processes for randomization that were either conducted through a third party or used a method that made knowledge of allocation impossible to predict, such as dynamic minimization.

#### 3.2.3. Measurement of the Outcome

All studies utilized Standardized Definitions for Efficacy Endpoints (STEEP) criteria to define DFS and OS [43].

#### 3.2.4. Selective Reporting

Of the seven RCTs included in the meta-analysis, one did not pre-specify the data analysis [33]. This resulted in moderate RoB for these studies; however, their reporting of results for DFS and OS did match the plan they outlined in their methods sections. One study [52] did not report the results from a multivariable Cox regression analysis of DFS despite conducting the analysis.

### 3.3. Effects of Interventions

#### 3.3.1. Disease-Free Survival

All seven studies included in the review reported DFS. This includes 11,803 participants, with 725 events in the anthracycline-taxane arm and 785 events in the TC arm. One of the studies pooled data from three separate trials—these data were reported and pooled individually [46]. Most studies reported DFS between 3–5 years. One study [51] reported DFS at 7 years. The RoB assessment for inconsistency and indirectness is small, but the RoB is relevant in the imprecision category (Figure 2). Heterogeneity across all studies is likely not important (I^2^ = 0%).

**Figure 3:**
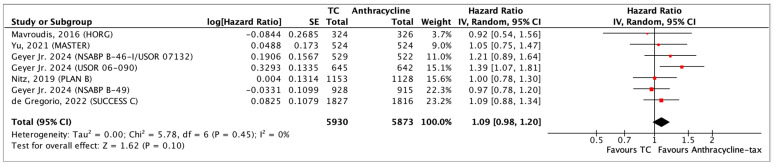
Forest plot of DFS comparing TC and anthracycline-taxane chemotherapy [33,46,51,52,54,55].

With a median follow-up of 60 months, TC chemotherapy showed little to no difference in DFS when compared to anthracycline-taxane chemotherapy (HR 1.09, 95% CI 0.98–1.20; *p* = 0.10; moderate certainty of evidence) (Figure 3). This value crosses the line of no effect as well as the minimum number for non-inferiority, based on the most conservative estimate in the included trials (Appendix A—Table A1—GRADE Summary: DFS). Using a baseline risk obtained from the included studies, for every 1000 patients who received chemotherapy, there were 123 disease recurrence events in the anthracycline-taxane and 133 in the TC group (Appendix A—Figure A1).

#### 3.3.2. Overall Survival

The pooled analysis from seven RCTs with OS data includes 11,803 patients and has a median follow-up of 60 months. There were 411 and 400 deaths in the TC and in anthracycline-taxane chemotherapy groups, respectively. One published paper pooled data from three separate trials—these data were reported and pooled individually [46]. The heterogeneity across all the studies is likely not more than what is due to chance (I^2^ = 0). The RoB was small for inconsistency, indirectness and imprecision categories.

**Figure 4:**
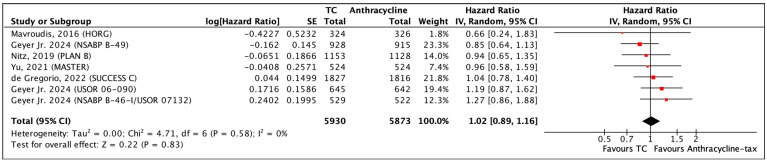
Forest plot of OS comparing TC and anthracycline-taxane chemotherapy [33,46,51,52,54,55].

The time-to-event outcome OS shows an HR of 1.02 (95% CI 0.89–1.16; *p* = 0.83) (Figure 4). In the quality of evidence assessment, no relevant difference in absolute risk of death events was found between the two treatments. Regarding the absolute treatment effect, no significant differences were found between the two treatment regimens, where there were 68 events of death for every 1000 patients treated with anthracycline-taxane and 69 in those treated with TC (Appendix A—Table A1—GRADE Summary: OS; Appendix A—Figure A2). Overall, there is a high quality of evidence that there is little to no difference in OS when comparing TC and anthracycline-taxane chemotherapy.

#### 3.3.3. Cardiotoxicity

**Figure 5:**
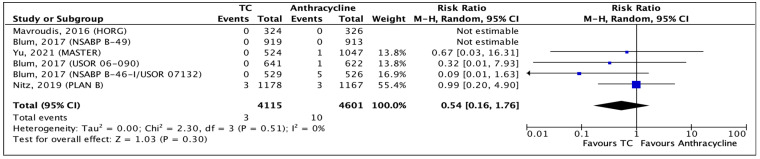
Forest plot of cardiotoxicity comparing TC and anthracycline chemotherapy [33,46,51,55].

All seven RCTs reported cardiotoxicity. All studies followed the CTAE guidelines for grading and reporting cardiotoxicity as HF [45]. One study [33] was comprised of three study arms, two being with anthracycline and one with TC chemotherapy. When pooling cardiotoxicity data, both anthracycline arms were combined for events and the total number of participants. From the total of 9732 treated patients, there were a total of thirteen events of cardiac-related side effects. Among those, 3 events are seen in the TC group and 10 in the anthracycline arm, with an RR of 0.54 (95% CI 0.16–1.76; *p* = 0.30) (Figure 5). Although the CI crosses the line of no effect, the absolute treatment effect demonstrates that for every 1000 breast cancer patients, two events of cardiotoxicity are likely to occur among those treated with anthracyclines and one among those treated with TC (Appendix A—Figure A3). The heterogeneity across all studies is likely not more than what is due to chance (I^2^ = 0%). In all categories, the RoB was not relevant. Overall, there is a high quality of evidence that there is a substantial difference between cardiotoxicity events when comparing TC and anthracycline chemotherapy, although this is not statistically significant.

### 3.4. Sub-Group Analysis

#### 3.4.1. ER Status

**Figure 6:**
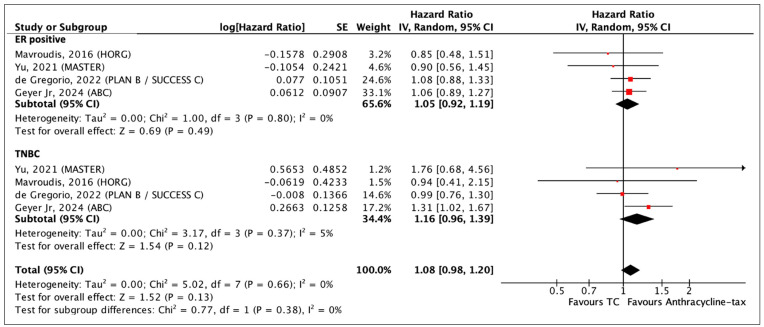
Forest plot of DFS comparing TC and anthracycline-taxane chemotherapy in a subgroup analysis of ER status [33,46,51,54].

There were 9052 patients who had luminal ER-positive breast cancer subtype and 2723 patients with TNBC. There is no significant difference in DFS between TC and anthracycline-taxane in the subgroup analysis of the ER-positive (HR 1.05, 95% CI 0.92–1.19; *p* = 0.49) and TNBC groups (HR 1.16, 95% CI 0.96–1.39; *p* = 0.12) (Figure 6). In the quality of evidence assessment, the heterogeneity across all the studies is likely not more than what is due to chance (I^2^ = 0% in ER-positive; I^2^ = 5% in TNBC).

#### 3.4.2. Lymph Node Status

**Figure 7:**
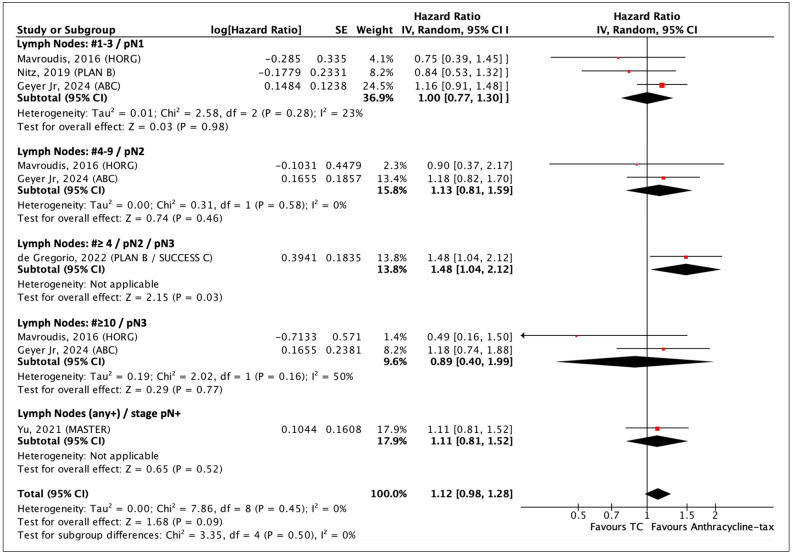
Forest plot of DFS comparing TC and anthracycline-taxane chemotherapy in a subgroup analysis of lymph node-positive [33,51,52,54,55].

Five studies reported on breast cancer patients with lymph node (LN)-negative disease. No difference in DFS was identified between anthracycline-taxane and TC chemotherapy (HR 1.06, 95% CI 0.87–1.28; *p* = 0.58), with no significant heterogeneity (I^2^ = 0%, *p* = 0.77) (Appendix A—Figure A4). In those who had LN involvement, there were no significant differences in the incidence of DFS events between anthracycline-taxane and TC within the following subgroups: LN #1–3 (pN1), LN #4–9 (pN2) and LN #≥10 (pN3). Only two studies combined the analysis on stages pN2 and pN3, which corresponds to LN #≥4. In this subgroup, anthracycline-taxane was, in fact, associated with reduced breast cancer recurrence compared with TC (HR 1.48, 95% CI 104–2.12; *p* = 0.03). However, the overall incidence of cancer relapse in patients who had LN-positive disease did not differ significantly between the anthracycline-taxane and TC groups (HR 1.12, 95% CI 0.98–1.28; *p* = 0.09; seven studies). Heterogeneity is not observed (I^2^ = 0%, *p* = 0.45) in Figure 7. Similarly, the OS analysis did not show the benefit of anthracycline-taxane over TC in women with LN-positive disease (HR 1.08, 95% CI 0.88–1.33; *p* = 0.47; five studies). No relevant heterogeneity is noticed across all the RCTs (I^2^ = 0%, *p* = 0.96) (Appendix A—Figure A5).

## 4. Discussion

Our work is an updated systematic review and meta-analysis on relevant RCTs published after February 2019 (the literature search dated 15 June 2018) comparing TC and anthracycline-taxane-based chemotherapy in stages I–III, high-risk, HER2-negative breast cancer patients.

We found that TC results in little to no difference in DFS or OS compared to anthracycline-taxane chemotherapies. These findings were consistent with moderate-to-high-quality evidence. Anthracycline-taxane chemotherapy regimens are the standard of care, with the use of TC as an alternative depending on certain contraindications. Therefore, with regard to treatment efficacy, our review does not support a change in the current standard of practice. In each case, specific patient circumstances need to be considered.

Our findings are consistent with a previous systematic review and meta-analysis by Caparica et al., 2019 which compared TC versus anthracycline-containing chemotherapy [39]. Their study revealed a similar magnitude of the effect between the two treatments. However, that study was published in 2019 and did not include Yu et al., 2021 [33], de Gregorio et al., 2022 [54] (updated results of the SUCCESS C and Plan B trials) or Geyer Jr. et al., 2024 [55] (updated results of the Anthracyclines in Early Breast Cancer (ABC) trials). They also included one study that recruited patients with any HER2 status, the DBCG 07-READ trial [56]. Our study expands upon these findings by including more recently published data and focusing exclusively on HER2-negative breast cancer patients treated with TC versus anthracycline-taxane chemotherapy rather than anthracycline-based regimens without taxane.

The ABC trials, containing three out of seven RCTs included in our study, the USOR 06-090, NSABP B-46-I/USOR 07132 and NSABP B-49 trials, recently reported a joint efficacy analysis. Their study showed, at 5 years, no OS differences between TC chemotherapy and anthracycline-taxane HR of 1.05 (95% CI 0.87–1.26; *p* = 0.64). Nonetheless, those treated with anthracycline-taxane had significantly fewer invasive DFS events, an HR of 1.38 (95% CI 1.16–1.65; *p* = 0.0003) and statistically increased rates of non-breast cancer mortality when compared with TC. Although death and breast cancer relapse were both included as invasive DFS events, the precise influence of non-breast cancer mortality on DFS in the ABC trials is not known [55].

All trials included in this work had the interventional arm, TC chemotherapy, designed to be delivered in a total of six cycles. Hence, there is no heterogeneity in the TC regimen amid the studies. It is important to emphasize that there are no RCTs that compared four cycles (×4) versus six cycles (×6) of TC chemotherapy or TC × 4 (instead of TC × 6) versus anthracycline-taxane chemotherapy. A retrospective real-world study addressed the comparison between TC × 4 versus TC × 6 and showed no difference in relapse and death events between the two regimens, and those treated with TC × 4 experienced fewer treatment toxicities [57]. The US Oncology 9735 trial compared TC × 4 versus the chemotherapy regimen without taxane and the anthracycline and cyclophosphamide (AC) regimen and demonstrated that TC × 4 was significantly better, improving OS and DFS [32]. Based on these findings, numerous oncology guidelines utilize TC × 4 in the treatment of adjuvant breast cancer [58].

Regarding the efficacy between the anthracycline agents, epirubicin and doxorubicin, many studies have demonstrated that these are interchangeable, without affecting patient’s survival outcome. Similarly, among the taxanes, paclitaxel administered on a 2-week dose-dense schedule versus paclitaxel on a weekly schedule or docetaxel administered every 3 weeks have all shown to be comparable options [59].

Our subgroup analysis on ER-positive, TNBC and LN status did not show substantial differences in breast cancer recurrent events between anthracycline-taxane and TC chemotherapy regimens, except in the subset of patients with LN #≥4 (pN2/pN3), where anthracycline-taxane was associated with improved DFS over TC.

We also found high-quality evidence to suggest that there is no significant difference in cardiotoxicity between the two groups. This result was surprising, as anthracyclines are known to carry a risk of irreversible cardiomyopathy, and TC is sometimes chosen as an alternative for patients with underlying cardiac disease. Although we found no significant difference in cardiotoxicity between the two treatment regimens, the absolute treatment effect did show an increased likelihood of cardiotoxic events in the anthracycline arm. Overall, however, events were rare across the trials. Only five of the studies reported on cardiotoxicity at all, and within these, some studies reported HF or LVSD as separate events. It is possible, therefore, that participants could have been classified into multiple categories based on their symptoms, resulting in misclassification. Thus, given these limitations, these results should be treated with caution and further investigation is needed prior to changing clinical practice.

It has been previously reported that 6% of patients who received anthracyclines develop clinically overt cardiotoxicity, with up to 18% developing subclinical CHF [25]. Risk factors for developing cardiotoxicity include cumulative anthracycline dose, chest radiotherapy, African American ethnicity, diabetes, hypertension and severe comorbidities [25]. In HER2-positive breast cancer, the addition of trastuzumab plus/minus pertuzumab remarkably increases the risk of cardiac impairment in patients previously treated with anthracycline [25,26]. Numerous studies that investigated the likelihood of anthracycline-inducing cardiac dysfunction included patients treated with monoclonal antibodies against the HER2 domain, which could potentially overestimate the cardiotoxicity risk of anthracyclines.

To our knowledge, there are no systematic reviews or meta-analyses comparing cardiotoxicity between anthracycline-taxane versus non-anthracycline regimens in breast cancer without the use of anti-HER2 therapies.

Our review has some limitations. Not all trials provided information regarding the ER status of their patients or the use of endocrine therapy after adjuvant chemotherapy, which may have influenced the time-to-event outcomes. We limited our search to publications in English, which would have excluded non-English language research. There were some data still outstanding at the time of analysis—one study [54] was still awaiting final publication for the outcomes of interest. The data from this study were obtained from multiple sources, including the study protocol [60] and a pooled analysis of the quantitative data [54]. One study, which did not report an HR (95% CI) [53], was excluded from the meta-analysis. However, these study results were summarized narratively in conjunction with the results from the meta-analysis.

Among 11,803 participants, there were 2723 (23%) patients who had TNBC. TNBC has a poorer prognosis than ER-positive breast cancer [61]. While this could impact the survival among these patients, it was reassuring that our TNBC subgroup analysis was consistent with our findings from the overall study cohort—there was no difference in OS or DFS between patients receiving anthracycline-taxane versus TC chemotherapy.

Genomic expression assays (GEAs), such as the Oncotype DX^®^ or MammaPrint^®^, are used to stratify patients based on their risk of recurrence and select high-risk patients for whom adjuvant chemotherapy would offer benefits [62,63]. For example, the Rx-PONDER [62] and the MINDACT [63] trials demonstrated that postmenopausal women with LN #1–3 (pN1) and low genomic risk for recurrence could have adjuvant chemotherapy omitted [62,63]. Among the studies included in our meta-analysis, only the PLAN B and the three studies of the ABC trials described the enrollment of women who had intermediate and high scores on GEAs. However, the precise number of patients who had GEA testing is unknown. The other three RCTs included in this meta-analysis did not require their participants to undergo GEA testing. Hence, patients with a potential low genomic risk of recurrence, in whom chemotherapy has not been predicted to be advantageous [64,65], may have been recruited and treated with chemotherapy.

A diagnosis of cardiotoxicity induced by anthracyclines depends on the cardiovascular benchmark set. All the RCTs included in this meta-analysis were conducted before the 2022 European Society of Cardiology (ESC) guidelines, where it has been well-established that Cancer Therapy-related Cardiac Dysfunction (CTRCD) is not only associated with reduced LVEF but evaluating CHF symptoms is contributory [66]. Thus, studies conducted prior to the 2022 ESC guideline are likely to have underestimated the incidence of CTRCD. In addition, none of the included studies in this meta-analysis distinguished between early and delayed onset of cardiotoxicity.

It is important to highlight that all clinical trials included in our meta-analysis provided adjuvant radiotherapy in accordance with the prevailing guidelines at the time of the studies. Similarly, adjuvant endocrine therapy was given to patients with ER+ BC, as indicated.

Strengths of this study include a vast systematic review, following the PRISMA guideline, of RCTs published up to March 2024. Our research showed a moderate to high certainty of evidence across the outcomes included. All studies were open-label; however, this was unlikely to affect the results given the objective guidelines for outcome evaluation. Studies tended to be downgraded due to imprecision and results that crossed the line of no effect; however, this may not be relevant for our analysis as our intention was to determine if TC is non-inferior to anthracycline-taxane chemotherapy, which includes an outcome of no-effect. Future studies should better outline the non-inferiority analysis boundaries.

All studies included in this review combined anthracyclines with taxanes. We consider this homogeneity a strength of this paper, particularly given that anthracycline-taxane combination regimens are the standard of care in this breast cancer population.

## 5. Conclusions

This review indicates that TC is a reasonable alternative to anthracycline-taxane chemotherapy for stages I–III, high-risk, HER2-negative breast cancer, with no meaningful difference in DFS or OS. However, in women with four or more positive lymph nodes (pN2/pN3), anthracycline-taxane was associated with a substantial reduction in relapse events compared with TC. Further studies of breast cancer patients treated with chemotherapy after having undergone a gene signature diagnostic assay are needed to better determine the lenses of genomic expressions, of which breast cancer populations would benefit the most from de-escalating anthracycline chemotherapy.

**Table 1:** Summary of key clinical trials, systematic reviews and meta-analyses that investigated the role of TC versus anthracycline-taxane in HER2-negative breast cancer.

Randomized Controlled Trial	Eligibility Criteria	Study Population	Median Follow up	DFS (95% CI)	OS (95% CI)
**Geyer Jr. et al., 2024** [55]/**J. Blum et al., 2017 [46]** Analysis of 3 RCTs * (*n* = 4242) · TC (6 cycles): docetaxel 75 mg/m^2^, cyclophosphamide 600 mg/m^2^, q3 weeks · TaxAC: doxorubicin 50 mg/m^2^, cyclophosphamide 500 mg/m^2^, q2–3 weeks, for four cycles, followed by paclitaxel (80 mg/m^2^ weekly (×12) or 175 mg/m^2^ q2 weeks for four cycles	If pN+: pT_any_ If pN_0_: > pT_2_ or TNBC. If pT_1c_ pN_0_ and ER+: grade 3 or high-risk score on Oncotype DX	ER+: 69% ER/PR−: 31% LN 0: 41% LN+_1–3_: 44% LN+_≥4_: 16%	3.3 years and 6.9 years (updated analysis)	5-year DFS TC: 85.1% TaxAC: 86.7% HR 1.14 (0.99–1.32), *p* = 0.08	5-year OS TC: 234 deaths TaxAC: 221 deaths HR 1.05 (0.87–1.26), *p* = 0.64)
**De Gregorio et al., 2022** [54] (*n* = 3643) · TC (6 cycles): docetaxel 75 mg/m^2^, cyclophosphamide 600 mg/m^2^ q3 weeks · FEC-D: 5-fluorouracil 500 mg/m^2^, epirubicin 100 mg/m^2^, cyclophosphamide 500 mg/m^2^, q3 weeks for three cycles, followed by docetaxel 100 mg/m^2^ q3 weeks for three cycles	Stages I–III high risk (pN+) or if pN0: ≥ pT_2_, grade 3, age ≤ 35 years, ER– PLAN B trial: All patients were offered OncotypeDX	ER +: 76.4% Luminal A: 53.4% Luminal B: 23% TNBC: 23.6%	5 years	5-year DFS rate: TC: 89.3% FEC-D: 90% TC vs. FEC-D: HR 1.05 (0.89–1.23), *p*= 0.565	5-year OS rate: TC: 94.9% FEC-D: 95% TC vs. FEC-D: HR 1.0 (0.79–1.25), *p*= 0.997
**Yu et al., 2021** [33] (*n* = 1571) · TC (6 cycles): docetaxel 75 mg/m^2^, cyclophosphamide 600 mg/m^2^ q3 weeks · FEC-D: fluorouracil 500 mg/m^2^, epirubicin 100 mg/m^2^ and cyclophosphamide 500 mg/m^2^, q3 weeks for three cycles, followed by docetaxel 100 mg/m^2^ every 3 weeks, for three cycles ·EC-P: epirubicin 90 mg/m^2^, cyclophosphamide 600 mg/m^2^,q3 weeks for 3 cycles, followed by weekly (×12) paclitaxel 80 mg/m^2^	pT_1–4_, pN+ or pT_2–3_, pN_0_ but high-risk (grade 2–3, age ≤ 35 years, or ER–)	Luminal A: TC: 22.9% CEF-T: 19.1% EC-P: 22.3% Luminal B: TC: 69.5% CEF-T: 73.4% EC-P: 69.9%	5.5 years	5-year DFS rate: TC: 85.0% CEF-T: 85.1% EC-P: 85.9 TC vs. EC-P: HR 1.05 (0.79–1.39), *p* = 0.771	5-year OS rate: TC: 96.5% CEF-T: 94.9% EC-P: 95.4% TC vs. EC-P: HR 0.96 (0.58–1.59), *p* = 0.893
**H. Ishiguro et al., 2020** [53] (*n* = 195) · TC (6 cycles): docetaxel 75 mg/m^2^, cyclophosphamide 600 mg/m^2^ · FEC-TC: 5-fluorouracil (500 mg/m^2^), epirubicin (100 mg/m^2^), cyclophosphamide (500 mg/m^2^), q3 weeks for three cycles, followed by TC q3 weeks, for three cycles	Stages I–III (except pT_1a_ or pT_1b_	ER+: 100% LN−: 49.2% LN+: 50.8%	5.8 years	No differences in IDFS (*p* = 0.854) between the treatment groups	No differences in OS (*p* = 0.911) between the treatment groups
**U. Nitz et al., 2019 [52]** (*n* = 3198) · TC (6 cycles): docetaxel 75 mg/m^2^, cyclophosphamide 600 mg/m^2^, q3 weeks · EC-T: Epirubicin 90 mg/m^2^, cyclophosphamide 600 mg/m^2^ q3 weeks, for four cycles, followed by docetaxel 100 mg/m^2^, q3 weeks for four cycles	ER+: pT_1–4c_, any pN+ or If pN_0_: grade II-III, TNBC or age < 35	ER+: 81.8% ER−: 18.2% pN_0_: 58.8% pN_1_: 34% pN_2–3_: 7.2%	5 years	5-year DFS TC: 89.6% EC-T: 89.8% HR 1.00 (0.77–1.3)	5-year OS TC: 94.7% EC-T: 94.5% HR 0.94 (0.65–1.34)
**D. Mavroudis et al., 2016 [51]** (*n* = 650) · FEC-D: 5-fluorouracil 500 mg/m^2^, epirubicin 75 mg/m^2^, cyclophosphamide 500 mg/m^2^, q2 weeks for four cycles, followed by docetaxel 75 mg/m^2^, q2 weeks for four cycles · TC (6 cycles): docetaxel 75 mg/m^2^, cyclophosphamide 600 mg/m^2^, q3 weeks	pT_1–4_, any pN+	ER+: 88% ER−: 11.4% Unknown: 0.6% LN+_1–3_: 63.7% LN+_≥4_: 33.7%	3.8 years	Median not reached HR 1.15 (0.71–1.84), *p* = 0.568 3-year DFS rate: FEC-D: 89.5% TC: 91.1%	Median not reached HR 1.16 (0.49–2.72), *p* = 0.738

RCT: randomized controlled trial; ER: endocrine receptor; TNBC: triple-negative breast cancer; LN: lymph node; * USOR 06090; NSABP B-46-I/07132; NSABP B-49.

## Figures and Tables

**Figure A1:**
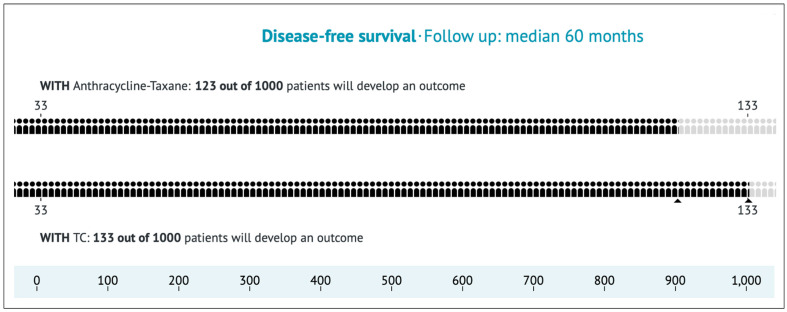
Baseline risk estimation of DFS events.

**Figure A2:**
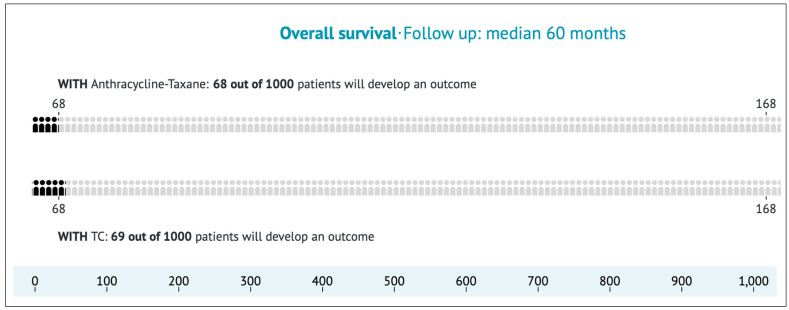
Baseline risk estimation of OS events.

**Figure A3:**
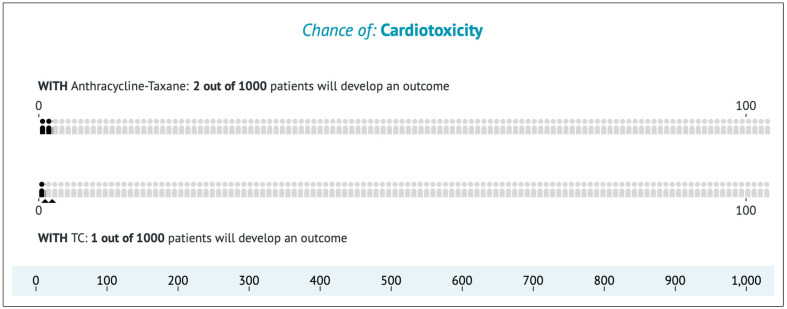
Baseline risk estimation of cardiotoxicity.

**Figure A4:**
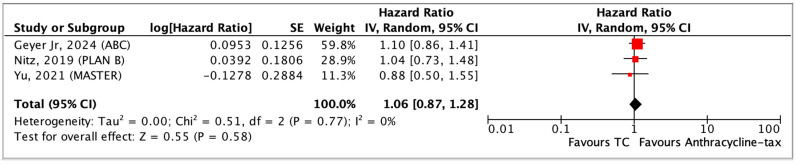
Forest plot of DFS comparing TC and anthracycline-taxane chemotherapy in a subgroup analysis of lymph node-negative [33,52,55].

**Figure A5:**
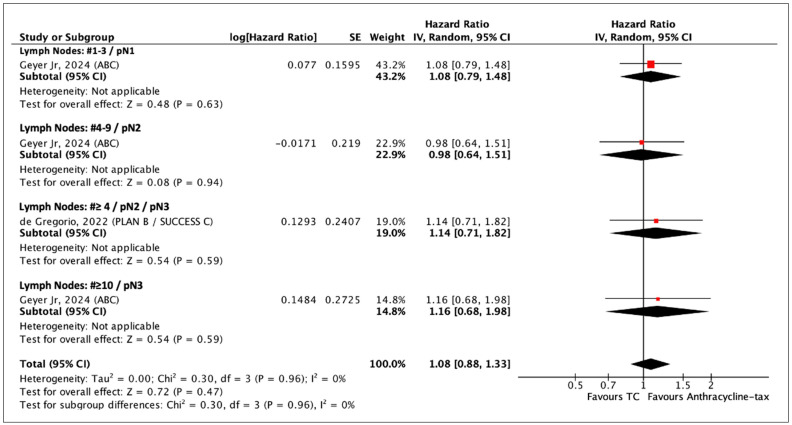
Forest plot of OS comparing TC and anthracycline-taxane chemotherapy in a subgroup analysis of lymph node-positive [54,55].

## Appendix A

Figure A1: Baseline risk estimation of DFS events; Figure A2: Baseline risk estimation of OS events; Figure A3: Baseline risk estimation of cardiotoxicity; Figure A4: Forest plot of DFS comparing TC and anthracycline-taxane chemotherapy in a subgroup analysis of lymph node-negative; Figure A5: Forest plot of OS comparing TC and anthracycline-taxane chemotherapy in a subgroup analysis of lymph node-positive; Table A1: Summary of findings. GRADE Evidence Profile.

**Table A1:** Summary of findings. GRADE Evidence Profile.

Certainty Assessment							№ of Patients		Effect		Certainty	Importance
№ of Studies	Study Design	Risk of Bias	Inconsistency	Indirectness	Imprecision	Other Considerations	TC	Anthracycline-Taxane	Relative (95% CI)	Absolute (95% CI)		
**Disease-free survival (DFS) (follow-up: median 60 months)**												
7	Randomized trials	Not serious	Not serious	Not serious	Serious ^a^	None	785/5930 (13.2%)	725/5873 (12.3%)	HR 1.09 (0.98 to 1.20)	10 more per 1000 (from 2 fewer to 23 more)	⨁⨁⨁◯ Moderate	CRITICAL
**Overall survival (OS) (follow-up: median 60 months)**												
7	Randomized trials	Not serious	Not serious	Not serious	Not serious	None	411/5930 (6.9%)	400/5873 (6.8%)	HR 1.02 (0.89 to 1.16)	1 more per 1000 (from 7 fewer to 10 more)	⨁⨁⨁⨁ High	CRITICAL
**Cardiotoxicity**												
5	Randomized trials	Not serious	Not serious	Not serious	Not serious	None	3/4115 (0.1%)	10/4601 (0.2%)	RR 0.54 (0.16 to 1.76)	1 fewer per 1000 (from 2 fewer to 2 more)	⨁⨁⨁⨁ High	CRITICAL

CI: confidence interval; HR: hazard ratio; Explanation: ^a^: The 95% CI crosses decision-making thresholds by including the possibility of no meaningful benefit and a meaningful benefit in DFS.
